# Controlled Synthesis of Ultrathin PtSe_2_ Nanosheets with Thickness‐Tunable Electrical and Magnetoelectrical Properties

**DOI:** 10.1002/advs.202103507

**Published:** 2021-10-28

**Authors:** Huifang Ma, Qi Qian, Biao Qin, Zhong Wan, Ruixia Wu, Bei Zhao, Hongmei Zhang, Zucheng Zhang, Jia Li, Zhengwei Zhang, Bo Li, Lin Wang, Xidong Duan

**Affiliations:** ^1^ Hunan Key Laboratory of Two‐Dimensional Materials and State Key Laboratory for Chemo/Biosensing and Chemometrics College of Chemistry and Chemical Engineering Hunan University Changsha 410082 China; ^2^ Institute of Advanced Materials (IAM) Nanjing Tech University (NanjingTech) Nanjing 211800 China; ^3^ Department of Chemistry and Biochemistry University of California Los Angeles California 90095 United States; ^4^ Department of Applied Physics School of Physics and Electronics Hunan University Changsha 410082 China; ^5^ School of Physics and Electronics Central South University Changsha 410083 China

**Keywords:** 2D materials, carrier mobility, chemical vapor deposition, Kondo effect, negative magnetoresistance

## Abstract

Thickness‐dependent chemical and physical properties have gained tremendous interest since the emergence of two‐dimensional (2D) materials. Despite attractive prospects, the thickness‐controlled synthesis of ultrathin nanosheets remains an outstanding challenge. Here, a chemical vapor deposition (CVD) route is reported to controllably synthesize high‐quality PtSe_2_ nanosheets with tunable thickness and explore their thickness‐dependent electronic and magnetotransport properties. Raman spectroscopic studies demonstrate all *E_g_*, *A*_1_ *_g_*, *A*_2_ *_u_*, and *E_u_* modes are red shift in thicker nanosheets. Electrical measurements demonstrate the 1.7 nm thick nanosheet is a semiconductor with room temperature field‐effect mobility of 66 cm^2^ V^−1^ s^−1^ and on/off ratio of 10^6^. The 2.3–3.8 nm thick nanosheets show slightly gated modulation with high field‐effect mobility up to 324 cm^2^ V^−1^ s^−1^ at room‐temperature. When the thickness is over 3.8 nm, the nanosheets show metallic behavior with conductivity and breakdown current density up to 6.8 × 10^5^ S m^–1^ and 6.9 × 10^7^ A cm^−2^, respectively. Interestingly, magnetoresistance (MR) studies reveal magnetic orders exist in this intrinsically non‐magnetic material system, as manifested by the thickness‐dependent Kondo effect, where both metal to insulator transition and negative MR appear upon cooling. Together, these studies suggest that PtSe_2_ is an intriguing system for both developing novel functional electronics and conducting fundamental 2D magnetism study.

## Graphical abstract

Ultrathin 2D PtSe_2_ nanosheets with various thickness are synthesized by a CVD method. The thickness dependent electrical and magnetoelectrical properties suggest that PtSe_2_ is an intriguing platform for both developing novel functional electronics and conducting fundamental 2D magnetism studies. The existence of magnetic order in this intrinsically non‐magnetic system will define a new class of magnetic material and stimulate further study.

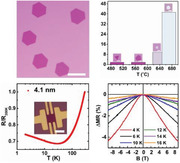

## 1 Introduction

2D layered atomic crystals have attracted extensive interest owing to their broad application potential in electronic, optoelectronic, catalytic, and magnetoelectronic devices.^[^ ^1^, ^2^, ^3^, ^4^, ^5^, ^6^, ^7^, ^8^, ^9^, ^10^, ^11^, ^12^, ^13^, ^14^, ^15^, ^16^, ^17^, ^18^ ^]^ The interaction between layers can dramatically affect the chemical and physical properties of 2D materials.^[^ ^19^ ^]^ In particular, the layer‐number dependent band gap in 2D materials offers new opportunities for their application in transistors, logic circuits, and photodetectors. For example, due to a thickness‐tunable bandgap change (0.3–2.0 eV from bulk to monolayer), black phosphorus is a promising optoelectronics material with a wide adsorption spectrum.^[^ ^20^, ^21^ ^]^ In addition, by reducing the bandgap to zero, metallic 2D materials can be realized. This has also attracted recent research interest in exploring multifunctional electrodes,^[^ ^22^ ^]^ superconductivity,^[^ ^14^, ^23^ ^]^ charge density wave,^[^ ^24^ ^]^ electro‐catalytic activity,^[^ ^25^ ^]^ and novel quantum phenomena.^[^ ^26^ ^]^ Therefore, the rich tunability of 2D materials provides an exciting platform for investigating novel functional electronic devices and condensed matter physics.

Platinum selenide (PtSe_2_) has a layered structure and changes from semiconductor to semimetal with increasing layer number.^[^ ^27^, ^28^ ^]^ The semiconducting PtSe_2_ is also predicted to have a carrier mobility of 2000 cm^2^ V^−1^ s^−1^ at room temperature,^[^ ^29^ ^]^ about six times higher than that of MoS_2_, which makes PtSe_2_ a promising candidate for developing 2D electronic and optoelectronic devices. The bulk PtSe_2_ is reported to be a type‐II Dirac semimetal, which may provide an important platform for exploring the exotic quantum phenomena.^[^ ^30^, ^31^ ^]^ On the other hand, in monolayer semiconducting PtSe_2_ film spin‐layer locking is observed, manifested as the local Rashba effect, which makes PtSe_2_ a great system for developing future spintronic devices.^[^ ^32^, ^33^ ^]^ It is also noted that PtSe_2_ nanosheets show air‐stability over one year.^[^ ^34^ ^]^ Additionally, 2D PtSe_2_ has promising application potential in sensors and photocatalysts for splitting water.^[^ ^35^, ^36^ ^]^ Thus, PtSe_2_ is an intriguing platform for both fundamental studies and technological development.

Although many potential research interests arose, the thickness‐tunable synthesis of high‐quality PtSe_2_ ultrathin nanosheets and systematic exploration of their thickness‐tunable electrical and magnetoelectrical properties remain a considerable challenge. Herein, we report a reliable CVD process for controllable growth of ultrathin PtSe_2_ nanosheets as thin as 1.7 nm. Optical microscopy (OM) and scanning electron microscopy (SEM) demonstrate that PtSe_2_ nanosheets show hexagonal or triangular shapes. X‐ray diffraction (XRD), transmission electron microscopy (TEM), selected area electron diffraction (SAED) and scanning transmission electron microscope (STEM) images show PtSe_2_ nanosheets are single crystals with excellent quality. Raman spectroscopy demonstrates that all *E_g_*, *A*_1_ *_g_*, *A*_2_ *_u_*, and *E_u_* modes are red shifted with increasing thickness. Transport studies display that PtSe_2_ nanosheets transform from semiconductor to metal when thickness increases, with strong thickness‐tunable electronic properties. Low temperature magnetotransport studies show evidence of the Kondo effect, suggesting the existence of magnetic moment in the intrinsically non‐magnetic material system. These studies demonstrate that 2D PtSe_2_ nanosheets have unique and excellent electrical properties, which may pave a promising opportunity for developing novel electronic and magnetoelectronic devices.

## 2 Results and Discussion

**Figure 1:**
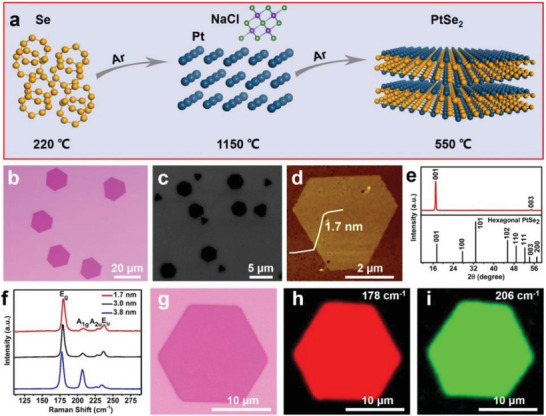
Synthesis and characterization of PtSe_2_ nanosheets. a) Schematic diagram of the synthesis of PtSe_2_ nanosheets. b) OM image of PtSe_2_ nanosheets. c) SEM image of PtSe_2_ nanosheets. d) AFM image of a 1.7 nm thick PtSe_2_ nanosheet. e) XRD pattern of PtSe_2_ nanosheets. f) Raman spectroscopic of PtSe_2_ nanosheets with different thicknesses. g) OM image of a hexagonal PtSe_2_ nanosheet and the corresponding (h, i) Raman mapping images with Raman peak located at 178 and 206 cm^–1^, respectively.

The PtSe_2_ nanosheets were successfully prepared by the CVD process at ambient pressure, with platinum powders and selenium power as precursors, NaCl as salt‐assisted precursors, and SiO_2_/Si as grown substrates (**Figure** **1**a). The typical OM and SEM images (Figure 1b,c, S1, Supporting Information) display PtSe_2_ nanosheets exhibit hexagonal shapes with highly uniform optical contrast and lateral size ranges from 1.8 to 36 µm. Atomic force microscopy (AFM) image reveals the resulted PtSe_2_ nanosheet with a thickness of down to 1.7 nm (Figure 1d). The XRD pattern also confirms the high‐quality single crystal nature of the resulting nanosheets with a hexagonal *P* $\bar{3}$ *m1* (164) space group (Figure 1e). The two main diffraction peaks correspond to the (001) and (003) planes of hexagonal PtSe_2_, demonstrating growth nanosheets with the direction of [001] family planes is perpendicular to the growth substrate.

The Raman spectroscopic of hexagonal PtSe_2_ nanosheet with two primary resonance peaks at around 180 and 208 cm^–1^ (Figure 1f), indexing to the in‐plane *E_g_* Raman active vibration mode and out‐of‐plane *A*_1_ *_g_* Raman active vibrational mode, respectively.^[^ ^37^ ^]^ The less prominent features at 225 and 235 cm^−1^ correspond to the *A*_2_ *_u_* and *E_u_* infrared active vibration modes, which are longitudinal optical modes attributed to the out‐of‐plane and in‐plane motions of Pt and Se atoms, respectively.^[^ ^37^ ^]^ Interestingly, we find when the material thickness increases from 1.7 nm to 3.8 nm, the *E_g_* and *A*_1_ *_g_* modes shift monotonically from 179.3 to 177.7 cm^–1^ and from 208.3 to 206.2 cm*^–^* ^1^, respectively. While meantime, the *A*_2_ *_u_* and *E_u_* modes shift from 228.5 to 225.7 cm*^–^* ^1^ and from 237.4 to 234.5 cm*^–^* ^1^, respectively. Thereby all *E_g_*, *A*_1_ *_g_*, *A*_2_ *_u_*, and *E_u_* modes display red shift in thicker materials. The tendency resembles the red shift of the *E* ^1^_2_ *_g_* peak of MoS_2_ and the *E*_g_, *A*_1_ *_g_* peaks of PtTe_2_ with increasing layer number.^[^ ^13^, ^38^ ^]^ Generally, the interlayer force suppresses atom vibration with increasing thickness, resulting in higher force constants.^[^ ^39^ ^]^ As a result, both *E* ^1^_2_ *_g_* and *A*_1_ *_g_* resonance peaks are supposed to show blue shift in thicker materials. Nevertheless, stacking‐induced structural changes or long‐range Coulombic interlayer coupling in PtTe_2_ and MoS_2_,^[^ ^13^, ^38^ ^]^ which could soften the vibration and develop a lower force constant, and as a result resonance peaks in thicker materials display red‐shifts. Figure 1g shows the OM image of a hexagonal PtSe_2_ nanosheet and Figure 1h,i displays the corresponding Raman intensity maps with exceedingly uniform color contrast across the entire domain, further confirming the uniformity of the resulting nanosheet.

**Figure 2:**
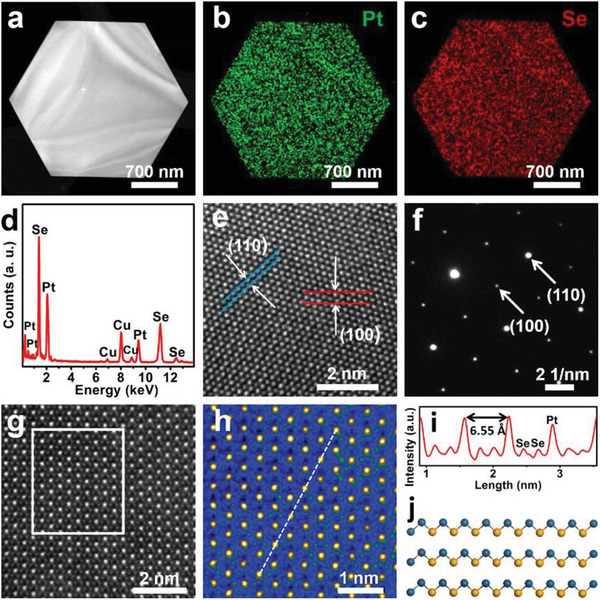
TEM and STEM characterizations of PtSe_2_ nanosheets. a) High angle annular dark field STEM (HAADF‐STEM) image of a PtSe_2_ nanosheet. b, c) EDS mapping pictures of a hexagonal PtSe_2_ nanosheet. d) EDS elemental analysis of a PtSe_2_ nanosheet. e, f) HRTEM and SAED images of PtSe_2_ nanosheets, respectively. g) STEM image of a PtSe_2_ nanosheet. h) False‐color STEM image in part g. i) Intensity line profile for the dashed line in picture g. j) Side view of PtSe_2_ crystal structure. Yellow and blue colors represent Pt and Se atoms, respectively.

**Figure** **2**a displays a HAADF‐STEM image of a PtSe_2_ nanosheet with a well‐faceted hexagonal geometry. Figure 2b,c show the corresponding EDS elemental mapping pictures with uniform contrast of Pt and Se elements, confirming the compositional uniformity of the resulting PtSe_2_ nanosheet. Figure 2d shows that EDS elemental analysis spectra of the resulting nanosheets demonstrates that the stoichiometric ratio Pt/Se is nearly 1:2. Lattice resolved TEM image exhibits a hexagonal lattice arrangement with the lattice spacings of 0.189 and 0.324 nm, which attribute to the (110) and (100) planes of the PtSe_2_ hexagonal structure, respectively (Figure 2e). The SAED pattern in Figure 2f further demonstrates that the resulting PtSe_2_ nanosheet is a high‐quality single crystal with a hexagonal structure. The resulting PtSe_2_ nanosheet is further characterized by STEM (Figure 2g), with Pt (white spots) and Se (gray spots) atoms clearly distinguished by the sharp contrasts generated by distinct atomic numbers. Figure 2h displays the false‐color STEM image of Figure 2g, where Pt and Se atoms could be more distinctly identified as yellow and green balls, respectively. Figure 2i shows the intensity line profile of Figure 2h, from which the lattice constant (*a*) of the grown sample is calculated to be ≈3.73 Å (6.55/√3). In general, these studies are very consistent with hexagonal PtSe_2_ crystals (Figure 2j).

**Figure 3:**
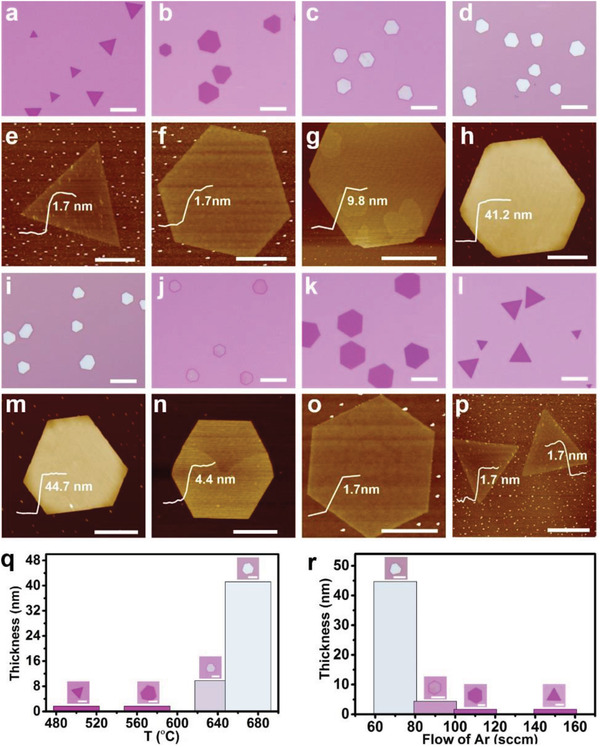
Thickness‐and morphology‐controlled growth of PtSe_2_ nanosheets on SiO_2_/Si. a–d) The temperature of central heating zone (1070 °C) and Ar flow rate (100 sccm) and obtainment of nanosheets with variable average thickness under different substrate temperatures. The substrate temperature in images (a–d) are ≈500, ≈570, ≈640, and ≈670 °C, respectively, and the e–h) corresponding representative AFM images, respectively. The scale bars in images (a–d) are 10 µm, in images (e–g) are 2 µm, in image (h) is 2 µm. i–l) The temperature of the central heating zone (1150 °C), the substrate temperature of ≈560 °C, and variable Ar flow rates can lead to nanosheets with different average thicknesses. The flow of Ar in images (i–l) is ≈70, ≈90, ≈110, and ≈150 sccm, respectively, and the m–p) corresponding representative AFM images, respectively. The scale bars in images (i–l) are 10 µm, in images (m, n) are 2 µm, in image (o) is 4 µm, in image (p) is 10 µm. q) Average thickness of PtSe_2_ at different substrate temperatures. Insets are OM pictures of the corresponding average thickness of PtSe_2_ nanosheets, all scale bars: 5 µm. r) Average thickness of PtSe_2_ nanosheets at different flow of Ar. Insets are OM images of the corresponding average thickness of PtSe_2_ nanosheets, all scale bars: 5 µm.

To controllably grow PtSe_2_ nanosheets with a tunable thickness, we have systematically explored the influence of substrate temperature and Ar flow rate (**Figure** **3**). We observed three main trends. First, the layer number and average lateral size of PtSe_2_ nanosheets increase with increasing substrate temperature (500–670°C) (Figure 3a–h, 3q, S3a, Supporting Information). Second, the layer number and average lateral size of PtSe_2_ reduces with increasing Ar flow rate (70–150 sccm) (Figure 3i–p, 3r, S3b, Supporting Information). Third, the PtSe_2_ domain morphology evolves from triangular towards hexagonal morphology with increasing substrate temperature or decreasing Ar flow rate. In general, at lower substrate temperatures, the nanosheet growth is controlled by edge energetics, with the precursor atoms quickly expanding to the fastest growth front to produce thinner nanosheets. Nonetheless, the growth is more dominated by thermodynamic control at elevated temperatures, resulting in thicker nanosheets with an overall smaller surface energy.^[^ ^40^, ^41^ ^]^ On the other hand, the growth at the high Ar flow rates is more kinetically governed, which shows a similar tendency to the CVD growth of VSe_2_, NiTe_2_.^[^ ^16^, ^42^ ^]^ In addition, the thickness distribution histograms of the PtSe_2_ nanosheets obtained at different substrate temperatures or Ar flow rates show a highly uniform thickness distribution, respectively, as shown in Figure S4a–d, S4e–h, Supporting Information.

**Figure 4:**
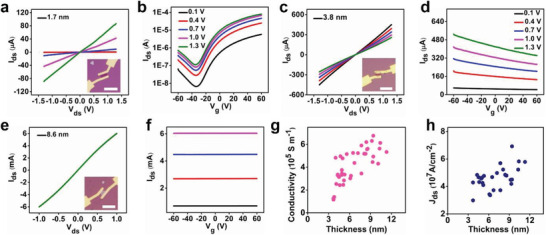
Electrical characterization of PtSe_2_ nanosheets. a, b) Output and transfer characteristics of a 1.7 nm thick PtSe_2_ nanosheet, respectively. The inset illustrates the OM picture of a 1.7 nm thick PtSe_2_ transistor. Scale bar: 10 µm. c, d) Output characteristics and transfer characteristics of a 3.8 nm thick PtSe_2_ nanosheet, respectively. The inset shows the OM picture of the 3.8 nm thick PtSe_2_ transistor. Scale bar: 10 µm. e, f) Output characteristics and transfer characteristics of an 8.6 nm thick PtSe_2_ nanosheet, respectively. The inset displays the OM picture of the 8.6 nm thick PtSe_2_ transistor. Scale bar: 10 µm. g) Thickness‐dependent conductivity for various thickness PtSe_2_ nanosheets. h) The thickness‐dependent breakdown current density for different thickness PtSe_2_ nanosheets.

We have further investigated the thickness‐dependent electrical performance of the resulted nanosheets by fabricating PtSe_2_ transistors on a SiO_2_/Si substrate with a bottom gate. **Figure** **4**a shows the output characteristics (*I*_ds_ vs. *V*_ds_) of a 1.7 nm thick PtSe_2_ nanosheet, which exhibits an on‐state current of 31 µA µm^−1^ at *V*_ds_ = ±1.3 V and *V_g_* = 60 V. The transfer characteristics of this 1.7 nm thick PtSe_2_ nanosheet show typical semiconductor behavior (Figure 4b), with a calculated field‐effect mobility of 66 cm^2^ V^–1^ s^–1^ (*L* = 2.48 µm, *W* = 4.35 µm, *V*_ds_ = 0.1 V). Figure 4c shows the output curves (*I*_ds_ vs. *V*_ds_) of a 3.8 nm thick PtSe_2_ nanosheet. The transfer characteristics of this 3.8 nm thick PtSe_2_ nanosheet resemble semimetallic behavior (Figure 4d). The calculated field‐effect mobility of the 3.8 nm thick PtSe_2_ nanosheet is 324 cm^2^ V^–1^ s^–1^ (*L* = 2.03 µm, *W* = 2.52 µm, *V*_ds_ = 0.1 V), which is about twice that reported in the exfoliated PtSe_2_ flakes.^[^ ^34^ ^]^ Figure 4e shows linear and symmetric output curves of an 8.6 nm thick PtSe_2_ nanosheet with little dependence on gate bias, resembling the typical behavior of a metal. And the flat transfer curves (*I*_ds_ vs. *V*_g_) further confirm the metallic property of the resulting nanosheet (Figure 4f). The conductivity of PtSe_2_ nanosheets varies from 1.3 × 10^5^ S m^−1^ at ≈3.7 nm thick to 6.8 × 10^5^ S m^−1^ at ≈9.4 nm thick at room temperature and also exhibits robust thickness‐dependent conductivity values (Figure 4g), comparing with exfoliation graphene,^[^ ^43^ ^]^ CVD growth of CoTe_2_,^[^ ^15^ ^]^ PtTe_2_^[^ ^13^ ^]^ and so on.

We have also explored the breakdown current density of the PtSe_2_ nanosheets. To this end, we increased the bias voltage on the transistor continuously until a sudden reduction of the current to zero was observed (Figure S2, Supporting Information). Here we refer to the current density right before the sharp reduction in current as the breakdown current density. Figure 4h displays the breakdown current density of PtSe_2_ nanosheets with the strong thickness dependence and the highest breakdown value up to 6.9 × 10^7^ A cm^−2^ at 9.4 nm thick, higher than that of CVD growth CoTe_2_,^[^ ^15^ ^]^ PtTe_2_,^[^ ^13^ ^]^ NiTe_2_,^[^ ^16^ ^]^ and so on.

**Figure 5:**
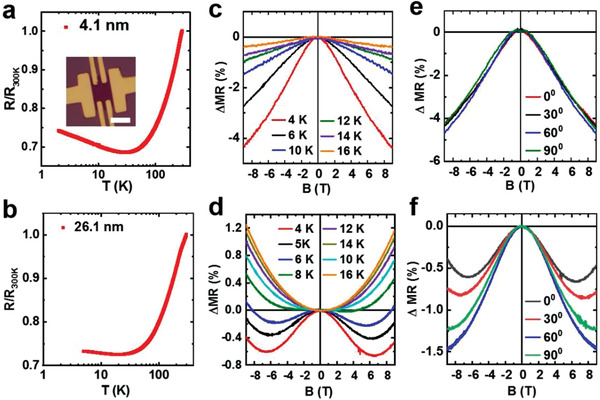
Low temperature magneto resistance study on PtSe_2_ Hall bar devices. a, b) Temperature‐dependent PtSe_2_ four‐terminal resistance with a thickness of 4.1 nm (a) and 26.1 nm (b), respectively. The inset in (a) illustrates the OM picture of a typical device used in this study, scale bar: 10 µm. c, d) Magnetoresistance of 4.1 nm (a) and 26.1 nm (b) thick PtSe_2_ devices measured at different temperature. e, f) Angle dependent magnetoresistance of PtSe_2_ device with a thickness of 4.1 nm (e) and 26.1 nm (f) taken at 4 K. Here the angle is measured between the magnetic field and the electric field direction.

Finally, in order to investigate the carrier transport properties of PtSe_2_ nanosheets at different temperatures, four terminal measurements were performed using the standard low‐frequency lock‐in technique on Hall bar devices fabricated on SiO_2_/Si substrates. For a sample with a 4.1 nm thickness, we saw its resistance first decrease upon cooling, consistent with the typical behavior of a metal. Interestingly, the resistance reaches its minimum at around 40 K, and starts to increase when the system further cools down. Namely, a metal‐to‐insulator transition occurs upon cooling (**Figure** **5**a). It has been suggested that the Pt vacancy generates magnetic impurities in this material system.^[^ ^44^ ^]^ Therefore, the metal‐to‐insulator transition observed here can be attributed to the Kondo effect,^[^ ^45^ ^]^ where the interaction between electron and magnetic impurity causes electron location, and thus increases the sample resistance. The effect becomes more significant as temperatures decrease, we therefore observed the increased sample resistance with a decrease in temperature below 40 K. A similar effect can also be observed in the 26.1 nm thick material (Figure 5b), but is less significant as compared with the 4.1 nm case, indicating the less dominant role of magnetic impurity with increasing sample thickness.

The low temperature localization effect due to magnetic impurity scattering will be suppressed by the presence of an external magnetic field,^[^ ^45^ ^]^ as manifested by the negative MR in the lowest temperature magnetotransport studies (Figure 5c,d). Also, when the system warms up, the Kondo effect becomes less significant due to the increase in thermal noise, and therefore the negative MR is less apparent (Figure 5c,d). Additionally, we studied the angle‐dependent MR of PtSe_2_ nanosheets at 4 K. The total negative MR component of the 4.1 nm thick nanosheets is not related to the angle of the magnetic field (Figure 5e), further confirming the Kondo dominated regime in the thin sample.^[^ ^46^ ^]^ But the 26.1 nm thick nanosheet exhibits an angle‐dependent MR (Figure 5f), which is due to the mix between the Kondo insulator state and metallic state of the PtSe_2_ thick film. A similar trend can also be observed in the temperature dependent MR, where negative MR are always present across the studied temperature in the 4.1 nm thin device (Figure 5c), while the positive quadratic component is clearly visible at high temperature (>8 K) for the 26.1 nm thick device (Figure 5d). We note here that it is possible to further tune the Fermi level of this system using electrostatic gating, and construct a system with both the Kondo effect and chiral anomaly, which will offer a platform with even richer physics.^[^ ^47^ ^]^

## 3 Conclusion

In conclusion, the high‐quality ultrathin PtSe_2_ single crystals have been prepared on SiO_2_/Si substrate with thickness down to 1.7 nm. Raman spectroscopic studies demonstrate that all *E_g_*, *A*_1_ *_g_*, *A*_2_ *_u_*, and *E_u_* modes are red shift in thicker nanosheets. Electrical transport studies reveal that 2D PtSe_2_ nanosheets transit from semiconductor to metal with increasing thicknesses. The resulting nanosheets also exhibit strong thickness‐tunable electronic properties. In particular, the 3.8 nm thick nanosheet displays high carrier mobility at room‐temperature. The increment of resistance with decreasing temperature and negative MR demonstrates the evidence of the Kondo effect. Our work demonstrates that 2D PtSe_2_ has unique and excellent electrical properties, which makes it a promising 2D material for both investigating condensed matter physics and novel functional electronic devices.

## 4 Experimental Section

### Preparation of PtSe_2_ Nanosheets

2D PtSe_2_ nanosheets were prepared by the CVD process at ambient pressure on SiO_2_/Si substrates (Figure 1a). In brief, 45 mg platinum powders and 900 mg selenium powers as the precursors were put into the highest temperature area of and the upstream area of the furnace, respectively. 2 mg NaCl as salt‐assisted precursors were put into the ceramic boat with platinum powders at the same heating area. NaCl as salt‐assisted precursors can reduce the melting point of the platinum powders, more details in the Supporting Information. The SiO_2_/Si substrate was put into the downstream area of the furnace. Next, the quartz tube was purged with 300 sccm Ar for 2 min. Then, the furnace was raised to 1070 °C and kept the temperature constant for 30 min, and the carrier gas rate was kept at 95 sccm. Finally, PtSe_2_ nanosheets were obtained after the furnace was cooled down naturally.

### Sample Characterizations

The morphology and thickness of PtSe_2_ nanosheets were acquired by an optical microscope (DP27, OLYMPUS) and atomic force microscope (Bioscope system, BRUCKER). The structure of PtSe_2_ nanosheets was conducted by Raman spectroscopic (invia‐reflex, Renishaw), XRD (D8‐Advance, Bruker), TEM (JEM‐2100F, JEOL), and STEM (Titan Cubed Themis G2300).

### Device Fabrication and Characterization

Regarding the device fabrication, the poly (methyl methacrylate) (PMMA) layer was firstly spin‐coated (3500 rpm for 50 s) on top of the sample surface with SiO_2_/Si substrate. Next, the sample was baked at 130 °C for 5 min. Then, 10/50 nm Ti/Au electrodes were patterned on the sample with a thin PMMA layer by using electron‐beam lithography to define FET devices or Hall bar devices, and followed by an electron beam evaporation process. The field effect properties were characterized at the Lake Shore TTPX probe station. And the magnetotransport properties were investigated in a physical property measurement system (Quantum design Inc).

## Conflict of Interest

The authors declare no conflict of interest.

## Supporting information

Supporting Information

## Data Availability

Research data are not shared.
