# Supplementary material for: Controlled Synthesis of Ultrathin PtSe2 Nanosheets with Thickness‐Tunable Electrical and Magnetoelectrical Properties
Source: Adv Sci (Weinh). 2021 Oct 28;9(1):2103507. doi: 10.1002/advs.202103507 (PMC8728827; doi:10.1002/advs.202103507)
Supplement: Supplementary file 1 — Supporting Information [file ADVS-9-2103507-s001.pdf]

WILEY-VCH

© 2021 Wiley-VCH GmbH

ADVANCED SCIENCE
Open Access

Supporting Information

for *Adv. Sci.,* DOI: 10.1002/advs.202003507

Controlled Synthesis of Ultrathin PtSe2 Nanosheets with Thickness-Tunable Electrical and Magnetoelectrical Properties

*Huifang Ma, Qi Qian, Biao Qin, Zhong Wan, Ruixia Wu, Bei Zhao, Hongmei Zhang, Zucheng Zhang, Jia Li, Zhengwei Zhang, Bo Li, Lin Wang, Xidong Duan**

Supporting Information

Controlled Synthesis of Ultrathin $PtSe_2$ Nanosheets with Thickness-Tunable Electrical and Magnetoelectrical Properties

*Huifang Ma, Qi Qian, Biao Qin, Zhong Wan, Ruixia Wu, Bei Zhao, Hongmei Zhang, Zucheng Zhang, Jia Li, Zhengwei Zhang, Bo Li, Lin Wang, Xidong Duan**

The effect of NaCl during the $PtSe_2$ synthesis process

The effect of NaCl in the synthesis of $PtSe_2$ may be that the Pt powder is dissolved in the molten NaCl, making it easier to volatilize and increasing the rates of the reaction .The TMDCs based on Pt are very difficult to synthetize because Pt powder has high melting point and low vapour pressure, which results in very low mass flux and limits the reaction. Molten salts (such as NaCl, KCl) can raise mass flux by reducing the melting point of the metal precursor, thus increasing the rates of the chemical reaction.[1]

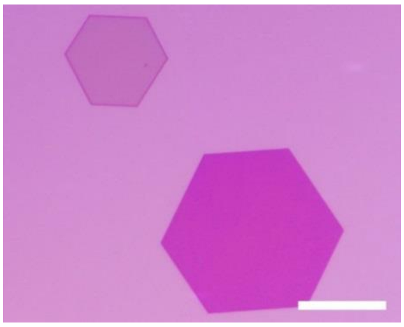

Figure S1. OM image of large size $PtSe_2$ nanosheets, scale bar: 20 μm.

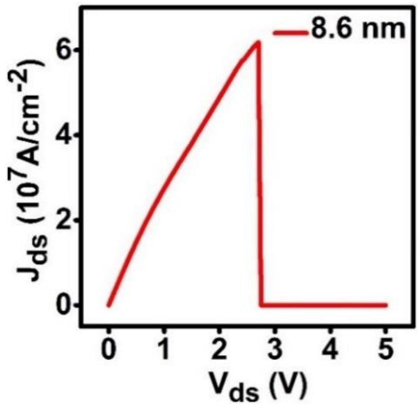

Figure S2. Breakdown current density measurement of an 8.6 nm thick $PtSe_2$ device.

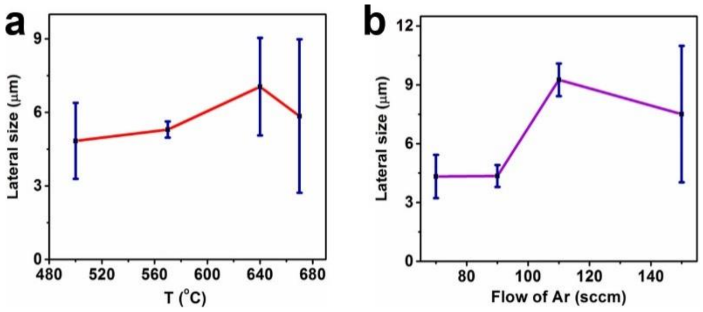

Figure S3. (a) Average lateral size of $PtSe_2$ at different substrate temperatures. The temperature of central heating zone (1070 °C) and Ar flow rate (100 sccm). (b) Average lateral size of $PtSe_2$ nanosheets at different Ar flow rates. The temperature of central heating zone (1150 °C), the substrate temperature of ~560 °C.

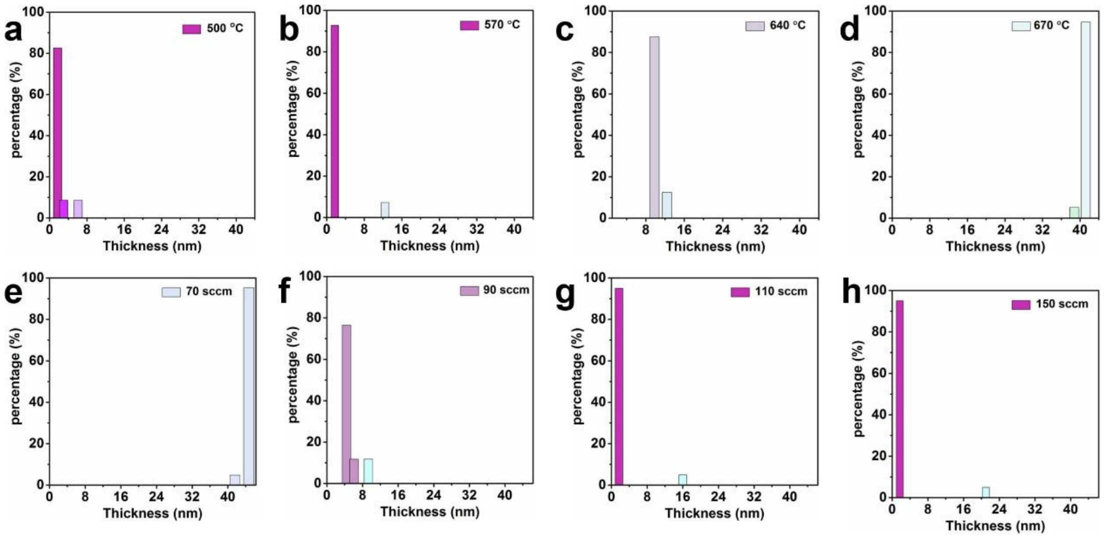

Figure S4. (a-d) The thickness distribution histograms of the $PtSe_2$ nanosheets obtained under different substrate temperatures, respectively. The temperature of central heating zone (1070 °C) and Ar flow rate (100 sccm). (e-h) The thickness distribution histograms of the $PtSe_2$ nanosheets obtained under different Ar flow rates. The temperature of central heating zone (1150 °C), the substrate temperature of ~560 °C.

We define the channel width W of the $PtSe_2$ nanosheets using the trapezoidal element current model[2]:

$$W = \frac{w_2 - w_1}{\ln w_2 - \ln w_1}$$

and Figure S5 shows the definition of w_1 and w_2. For a typical FET device (Figure 4a), L= 2.5 μm and W = 4.4 μm. And the electrode width is 2.1 μm. And for the typical device used for magnetoresistance studies (Figure 5a), the voltage electrodes are 2.7 μm in width, and the source-drain electrodes are 6.4 μm in width, the channel length L is 10.5 μm and channel width W is 16.1 μm.

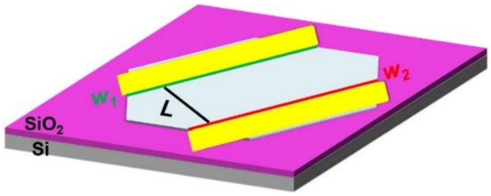

Figure S5. $PtSe_2$ nanosheet FET.

Table S1. Controlled synthesis of $PtSe_2$ nanosheets with different average thickness under different substrate temperatures.

Platinum powders temperature (ºC)	Selenium powders temperature (ºC)	Substrate temperature (ºC)	Ar flow rate (sccm)	Growth time (min)	Thickness (nm)
1070	250	670	95	25	~1.7
1070	250	640	95	25	~1.7
1070	250	570	95	25	~9.8
1070	250	500	95	25	~41.2

Table S2. Controlled synthesis of $PtSe_2$ nanosheets with variable average thickness under different Ar flow rates.

Platinum powders temperature (ºC)	Selenium powders temperature (ºC)	Substrate temperature (ºC)	Ar flow rate (sccm)	Growth time (min)	Thickness (nm)
1150	250	560	70	25	~44.7
1150	250	560	90	25	~4.4
1150	250	560	110	25	~1.7
1150	250	560	150	25	~1.7

References

[1] J. Zhou, J. Lin, X. Huang, Y. Zhou, Y. Chen, J. Xia, H. Wang, Y. Xie, H. Yu, J. Lei, D. Wu, F. Liu, Q. Fu, Q. Zeng, C.-H. Hsu, C. Yang, L. Lu, T. Yu, Z. Shen, H. Lin, B. I. Yakobson, Q. Liu, K. Suenaga, G. Liu, Z. Liu, *Nature* **2018**, *556*, 355.

[2] R. Giacomini, J. A. Martino, *J. Electrochem. Soc.* **2006**, *153*, G218.
